# Residue 365 in Hemagglutinin–Neuraminidase Is a Key Thermostable Determinant of Genotype VI.2.1.1.2.2 Newcastle Disease Virus

**DOI:** 10.3390/v17070977

**Published:** 2025-07-13

**Authors:** Tao Di, Ran Zhao, Qiankai Shi, Fangfang Wang, Zongxi Han, Huixin Li, Yuhao Shao, Junfeng Sun, Shengwang Liu

**Affiliations:** State Key Laboratory for Animal Disease Control and Prevention, Division of Avian Infectious Diseases, Harbin Veterinary Research Institute, The Chinese Academy of Agricultural Sciences, Harbin 150069, China; di_tao@126.com (T.D.); zhaoran@caas.cn (R.Z.); shiqiankai@caas.cn (Q.S.); wangffar@163.com (F.W.); hanzongxi@caas.cn (Z.H.); lihuixin@caas.cn (H.L.); shaoyuhao@caas.cn (Y.S.)

**Keywords:** Newcastle disease virus, genotype VI, hemagglutinin–neuraminidase, thermostability

## Abstract

Newcastle disease virus (NDV) genotype VI from pigeon origin is an important causative agent for serious disease in pigeons. Although the biological characteristics of genotype VI NDV have been extensively studied, the understanding of the thermostability of this genotype is still incomplete. In this study, an NDV strain, designated P0506, was isolated from a diseased pigeon in China and classified as genotype VI. Phylogenetic analysis on the basis of the Fusion gene coding sequence indicated that P0506 belonged to sub-genotype VI.2.1.1.2.2 of class II. The thermostability may be a universal characteristic of genotype VI NDV. Thus, the thermostability of two strains, including P0506 identified in this study and P0713 identified previously, belonging to VI.2.1.1.2.2, and another previously isolated strain, P0813, in VI.2.1.1.2.1, was investigated. It was indicated that all three viruses presented resistance to heat treatment, but P0713 was more robust than P0813 and P0506. By constructing a series of HN protein mutants, amino acid residues at both residues 365 and 497 in HN protein were found to be involved in the heat resistance. Furthermore, the effects of residues 365 and 497 in HN protein on the thermostability of the virus were further evaluated by using recombinant viruses generated by the reverse genetic system. Our results showed that residue at position 365 in HN protein was the key thermostable determinant of sub-genotype VI.2.1.1.2.2 NDV. These findings will help us better understand the thermostable mechanism of NDV and serve as a foundation for the further development of novel thermostable vaccines.

## 1. Introduction

Newcastle disease (ND) is an acute, highly contagious avian infectious disease caused by virulent strains of Newcastle disease virus (NDV) [1]. ND was first identified in Indonesia and England in the 1920s; since then, four major ND pandemics have occurred worldwide. Currently, this disease remains one of the most significant threats to the global poultry industry [2,3]. Since its first report, the genetic evolution of NDV has been occurring continuously. According to the latest classification system, NDV can be divided into two clades: class I and class II [4]. Class I NDV contains low-pathogenicity viruses isolated from wild birds and live poultry markets, within one genotype. Class II NDV includes a series of avirulent to virulent strains that have been isolated from both wild birds and domestic poultry and is classified into 20 genotypes [5,6].

Genotype VI NDV in class II, commonly referred to as Pigeon paramyxovirus type 1 (PPMV-1), results in ND-like disease in pigeons with high rates of morbidity and mortality [7,8]. PPMV-1 is primarily isolated from species of the *Columbidae* family and is considered to have originated from the host adaptation of old virulent NDV in pigeons. PPMV-1 was first described in the Middle East (Iraq) around 1978 [9,10]. Subsequently, this causative virus spread to Europe and then rapidly disseminated to other countries of the world, leading to the third global ND pandemic [11]. Genotype VI is the most diverse genetic group of NDV, and isolates in this genotype can be further assigned into seven sub-genotypes, including VI.1, VI.2.2.1, VI.2.2.2, VI.2.1.2, VI.2.1.1.1, VI.2.1.1.2.1, and VI.2.1.1.2.2 [4]. Based on the epidemiological surveillance data, isolates belonging to four sub-genotypes VI.1, VI.2.2.2, VI.2.1.1.2.1, and VI.2.1.1.2.2 have appeared in China [12,13,14].

In China, the ND incidence caused by genotype VII virus has remarkably declined due to the application of an inactivated vaccine containing genotype VII virus in chickens and geese [15,16]. However, with the rapid development of the pigeon industry, genotype VI viruses have been constantly isolated from pigeons in recent years. Since the ND outbreaks caused by the spread of genotype VI virus to chickens have been recorded [17,18,19], the prevalence of genotype VI viruses may also represent a potential threat to domestic poultry. However, the understanding of the biological characteristics of genotype VI NDV is limited.

Resistance to heat is one of the attractive biological characteristics of NDV, which may be attributed to the adaptation of NDV to environmental factors during dispersal. Though most of the NDV isolates are sensitive to heat, a few thermostable strains, such as V4, I-2, and K148/08 strains in genotype I and AF2240 and HR09 strains in genotype VIII, have been identified [20,21,22,23]. The thermostability of a virus can be utilized for the development of a thermostable vaccine, which is partially or completely independent of a cold chain. At present, commercial thermostable vaccines have been developed from V4 and I-2 and used to protect poultry flocks against NDV infection in some developing countries [1,24,25]. Investigation of the molecular basis of NDV thermostable phenotype using V4 and HR09 as models suggests that hemagglutinin–neuraminidase (HN) protein is the major contributor to NDV thermostability, and residues 315 and 369 in HN protein are the key determinants [23,26,27]. For genotype VI NDV, though thermostable isolates have been reported in Italy and Australia [28,29], the molecular mechanism that accounts for thermostability has not been disclosed.

In this study, the thermostability of representative strains from two recently prevalent sub-genotypes in China, VI.2.1.1.2.1 and VI.2.1.1.2.2, was evaluated. Then, a series of HN mutant proteins were constructed to screen the amino acids that may be involved in thermostability. Furthermore, recombinant viruses were generated using a reverse genetic system to verify the key amino acids that contributed to viral thermostability. Our results showed that glycine residue at position 365 in HN protein was the key thermostable determinant of sub-genotype VI.2.1.1.2.2 NDV. Our findings provide insights for understanding the thermostable mechanism of genotype VI NDV and the development of novel thermostable vaccines.

## 2. Materials and Methods

### 2.1. Cells and Eggs

Expi293F suspension cells were cultured in Expi293 Expression Medium (Thermo Fisher Scientific, Waltham, MA, USA) following the manufacturer’s instructions. BSR-T7/5 cells and DF-1 cells were cultured in Dulbecco’s modified Eagle’s medium (DMEM) containing 10% fetal bovine serum (FBS) (Sigma, Shanghai, China). Specific-pathogen-free (SPF) embryonated eggs were provided by Harbin Veterinary Research Institute, Chinese Academy of Agricultural Sciences (HVRI, CAAS).

### 2.2. Sample Collection and Virus Isolation

A genotype VI NDV strain was isolated from trachea samples from a diseased pigeon in Heilongjiang province in China in 2017. The pigeon from which samples were collected showed respiratory and neurological symptoms. Trachea sample was treated and inoculated into the allantoic cavity of three 9-day-old SPF embryonated chicken eggs as previously described [30]. Then, the allantoic fluids were harvested and subjected to hemagglutination (HA) and hemagglutination-inhibition (HI) assays with anti-genotype VI NDV (pi/CH/LHLJ/110822) chicken serum [31] and a panel of reference antisera against the 16 HA subtypes of avian influenza virus [32]. After three times of purification in primary chicken embryo fibroblasts, one NDV isolate was obtained and designated pi/CH/LHLJ/170506 (P0506).

### 2.3. Sequence and Phylogenetic Analysis

Viral RNA of P0506 was extracted from infectious allantoic fluid using TRIzol reagent (Invitrogen, Carlsbad, CA, USA) following the manufacturer’s instructions. The complete genome, including 3′/5′ terminal regions of P0506, was amplified and sequenced as previously described [30,33]. The genome sequence assembly, coding sequence (CDS) search, and sequence alignment were conducted with DNASTAR’s Lasergene Sequence Analysis Software version 7.1 (DNASTAR Inc., Madison, WI, USA) [34] and Geneious software Prime 2022.1.1 (Biomatters, Auckland, New Zealand) [35]. The complete genomic sequence of P0506 was deposited in GenBank with the accession number: PV837547. The complete F gene CDS of P0506 was combined with the class II pilot F gene dataset established by Dimitrov et al. for phylogenetic analysis [4]. A phylogenetic tree was constructed with MEGA X software using maximum likelihood (ML) with 1000 bootstraps. Deduced amino acid sequences of HN protein were downloaded from GenBank and subjected to comparative analysis by using Geneious software Prime 2022.1.1 (Biomatters, Auckland, New Zealand) [35].

### 2.4. Thermostability Test

Based on the result of phylogenetic analyses, our strain P0506 and the previously isolated strains pi/CH/LHLJ/110813 (P0813) in sub-genotype VI.2.1.1.2.1 and pi/CH/LLN/110713 (P0713) in sub-genotype VI.2.1.1.2.2 [30] were selected for further study. Thermostability of NDVs was determined as previously described [23,27]. Briefly, aliquots of virus-infected allantoic fluids were incubated in a water bath at 56 °C, triplicate samples of each virus were collected and then transferred to ice to stop the heat inactivation at 10 min intervals until 60 min after incubation. The HA activities and infectivity of heat-treated viruses were evaluated by HA assay and 50% tissue culture infectious dose (TCID_50_) assay in DF-1 cells as previously described [36].

### 2.5. Construction of Plasmids and Expression of HN Mutants

Based on the results from viral thermostability test, the CDS of HN gene of P0506 and P0713 were amplified from viral RNA and cloned into the pCAGGS vector. The constructed plasmids were designated pCAG-P0506-HN WT and pCAG-P0713-HN WT, respectively. Sequence alignment revealed that there were five different amino acids between HN proteins of P0506 and P0713, which were located at 73, 92, 266, 365, and 497 positions in HN protein, respectively. Hence, plasmids expressing S73L, S92F, A266T, S365G, and T497A mutant in P0506 HN proteins and plasmids expressing L73S, F92S, T266A, G365S, and A497T mutant in P0713 HN proteins were constructed via site-directed mutagenesis using specific primers, the resulting plasmids were designated pCAG-P0506-HN-S73L, -S92F, -A266T,-S365G, and -T497A, and pCAG-P0713-HN-L73S, -F92S, -T266A, -G365S, and -A497T, respectively.

Suspended Expi293F cells were transfected with above plasmids, respectively, using an ExpiFectamine293 Transfection Kit (Thermo Fisher Scientific, Waltham, MA, USA) in accordance with the manufacturer’s instructions. After 5 days, the cell culture supernatants were harvested, and HN proteins in the supernatants were purified as described previously [36]. The obtained proteins were quantified using the Bradford Protein Assay Kit (Beyotime, Shanghai, China) and examined by sodium dodecyl sulfate polyacrylamide gel electrophoresis (SDS-PAGE). The obtained wild-type HN proteins and corresponding mutants were heat-treated at 56 °C for different times, and then the HA titers of these proteins were determined via HA assay.

### 2.6. Recovery of P0506, P0173 and HN Mutant Recombinants from cDNA

The full-length cDNAs of P0506 and P0173 were cloned into the transcription vector pOLTV5, respectively, as previously described [33,37,38,39]. Briefly, for P0506, the pOLTV5 vector was amplified by PCR using primers containing 15 nt of the P0506 genomic 3′ and 5′-terminal sequences. The nucleotide fragments from 1 to 2760 and from 14,319 to 15,192 in the genome of P0506 were amplified and fused to generate a chimera containing a Pme I restriction site. Then the constructed chimera was cloned into pOLTV5 vector to generate a plasmid named P0506-1 using an In-fusion cloning kit (Clontech, Takara, Dalian, China). Subsequently, the fragments 2761-6089, 6090-8881, 8882-11675, and 11676-14318 in the genome of P0506 were amplified and cloned into the plasmid constructed in the previous step through the Pme I restriction site using the In-fusion cloning kit. The ultimate plasmid, which contained the full-length genome of P0506, was designated as P0506FL. For P0713, the fragments 1-2805 and 14319-15192 of the genome were amplified, fused, and cloned into pOLTV5 vector as described above. Then, the fragments 2806-5869, 5870-8881, 8882-11675, and 11676-14318 were successively cloned into the transcription vector to generate the plasmid containing the full-length genome of P0713, designated as P0713FL. Subsequently, A1093G and A1489G mutations in HN CDS in P0506FL and G1093A and G1489A mutations in HN CDS in P0713FL which resulting in 365 and 497 amino acid alterations, were performed using overlap PCR with specific primers, respectively. The resultant plasmids were designated as P0506FL-S365G, -T497A, P0713FL-G365S, -A497T, respectively.

Rescue of recombinant virus was performed by transfection of the plasmids P0506FL, P0506FL-S365G, -T497A, P0713FL, P0713FL-G365S, -A497T together with helper plasmids which code NP, P, and L proteins into BSR T7/5 cells following the previously described procedure [38,39]. After 48 h, the culture supernatant and cells were harvested and inoculated into the allantoic cavities of 9-day-old SPF embryonated chicken eggs. Virus particles in the allantoic fluid were confirmed via HA and HI assays with antibody against genotype VI NDV [31]. The rescued virus was designated as rP0506, rP0506-S365G, -T497A and rP0713, rP0713-G365S, -A497T, respectively. All rescued viruses were successively passaged three times, and the virus stocks were prepared by inoculating 9-day-old SPF embryonated chicken eggs. The genomic sequences of these rescued viruses were confirmed by sequencing.

### 2.7. Virus Titer and Thermostability of Recombinant NDVs

The titers of rP0506, rP0506-S365G, -T497A, and rP0713, rP0713-G365S, -A497T stocks were determined by the HA assay, the TCID_50_ assay on DF-1 cells, and the 50% egg infectious dose (EID_50_) assay in 9-day-old SPF embryonated chicken eggs, respectively. The thermostability of each virus was determined as described in Section 2.4.

### 2.8. Comparison of Thermostability of Different NDV Strains

The heat resistance of strain V4 in class II genotype I, strain La Sota in class II genotype II, and two class I strains, FJ28 and SH101, which identified in our previous work [33] were determined as described in Section 2.4 and the HA activities of heat-treated viruses were evaluated by HA assay.

### 2.9. Statistical Analysis

GraphPad Prism 5 Software (GraphPad, La Jolla, CA, USA) was adopted for statistical analysis. Data were expressed as means and standard deviations (SD). One-way analysis of variance with Bonferroni’s correction was employed for multigroup comparisons. The differences were considered significant if *p* values were < 0.05.

## 3. Results

### 3.1. Virus Isolation and Identification

SPF eggs inoculated with samples from a diseased pigeon died between 2 to 3 days post inoculation. The viruses in the allantoic fluid were identified by HA and HI assays. The HA titers of allantoic fluids harvested from three eggs ranged from 2^7^ to 2^8^, and their HA activity was specifically inhibited with anti-genotype VI NDV (pi/CH/LHLJ/110822) chicken serum at HI titers of 8 × log_2_. In contrast, the virus did not react with reference antisera against the 16 avian influenza virus subtypes.

### 3.2. Sequence and Genetic Analysis

The complete genomic sequence of P0506 was 15,198 nt in length, with the 3′ leader and 5′ trailer sequences being 55 and 114 nt, respectively. The genomic organization of P0506 followed the “rule of six”, containing six genes in the order 3′-NP-P-M-F-HN-L-5′, which was similar to those of other genotypes VI NDV. The amino acid motif at the cleavage site of the F protein of P0506 was ^112^RRQKRF^117^, which is characteristic of virulent NDV. The F protein contained six potential N-glycosylation sites located at positions 85, 191, 366, 447, 471, and 541, and the HN protein contained five potential N-glycosylation sites located at positions 119, 341, 433, 481, and 508.

**Figure 1:**
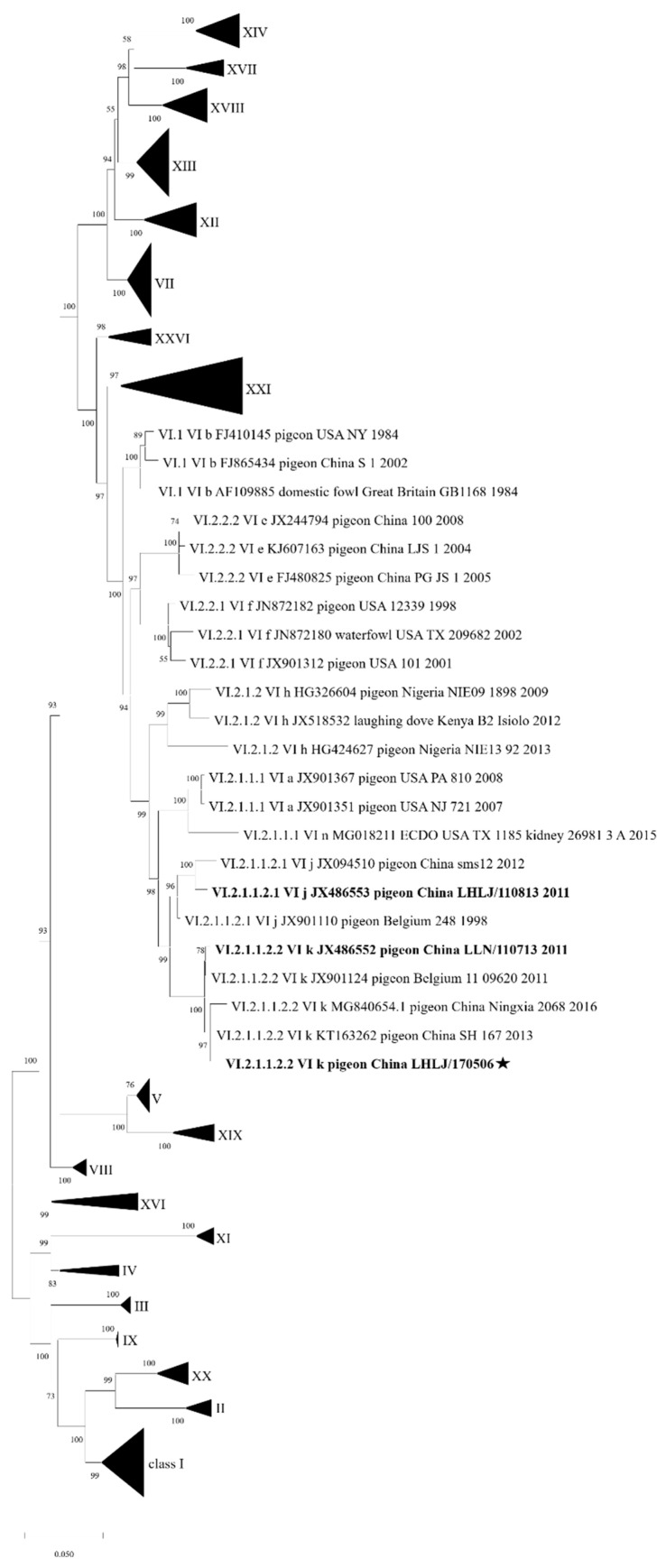
Phylogenetic tree constructed using the complete CDS of F gene of P0506 and other reference NDV strains. The F CDS of P0506, combined with the class II pilot F gene dataset established by Dimitrov et al., was subjected to phylogenetic analysis. A phylogenetic tree was constructed with MEGA X software using maximum likelihood (ML) with 1000 bootstraps. Except genotype VI, other genotypes were represented by compressed triangles. P0506 was labelled with a black pentastar.

In the phylogenetic tree (Figure 1), our P0506 was classified into sub-genotype VI.2.1.1.2.2, which contained P0713 [30] and two isolates in China and one isolate in Belgium. The strain P0713 was the first identified sub-genotype VI.2.1.1.2.2 isolate in China, which was the prevalent sub-genotype in pigeon populations in China since about 2011 [13]. Another strain, P0813, was classified as sub-genotype VI.2.1.1.2.1 in the phylogenetic analysis.

### 3.3. Signature Features of Amino Acid Residues at Positions 315 and 369 in HN Protein

**Figure 2:**
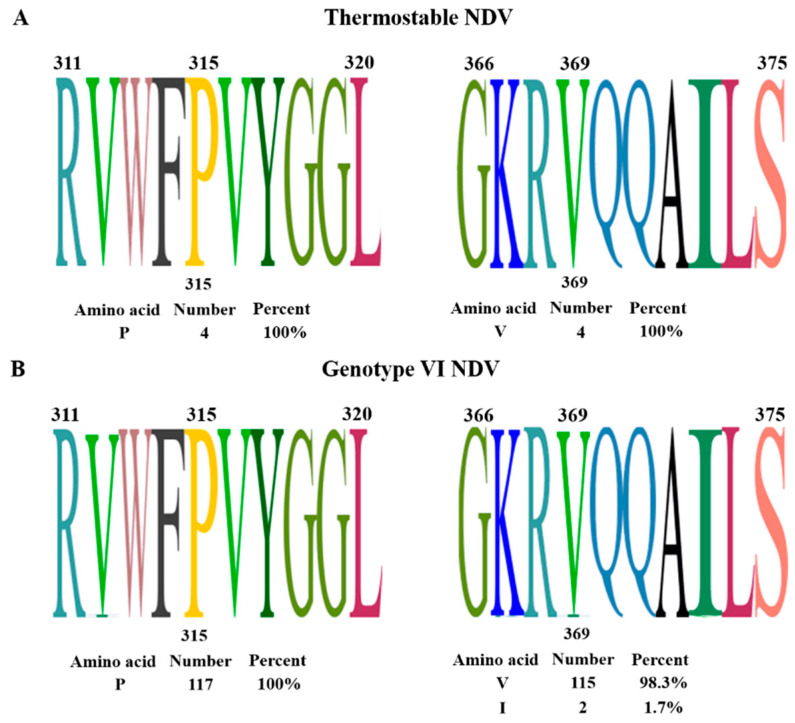
Signature features of amino acid residues at positions 315 and 369 in the HN protein. Amino acid residues flanking positions 315 and 369 in HN protein from 4 thermostable NDV strains (**A**) and 117 genotype VI isolates (**B**) were aligned. HN CDS sequences from 117 genotype VI strains deposited before April 18, 2025, were downloaded from GenBank. Type, number, and percentage of amino acid residues at positions 315 and 369 are shown.

It has been suggested that P315 and V369 in the HN protein of the thermostable strain HR09 significantly contributed to its thermostable phenotype [23]. In this study, the signature features of amino acid residues at positions 315 and 369 in HN protein from four known thermostable strains and 117 genotype VI NDV were analyzed. As shown in Figure 2A, the P and V amino acid residues at positions 315 and 369 were conserved in HN protein from thermostable strains V4, I-2, AF2240, and HR09, suggesting that these two amino acid residues might be potentially involved in the thermostability of NDV. Subsequently, the amino acid sequences of the HN protein from all available genotype VI NDV were further analyzed. Similar to the thermostable strains, the amino acid residue P at position 315 was found in the HN protein from all genotypes of VI NDV. In addition, amino acid residues V (98.3%) and I (1.7%) were found at the 365 position (Figure 2B). These results indicated the possibility of the thermostable phenotype of genotype VI NDV.

### 3.4. Identification of Viral Thermostable Phenotype

**Figure 3:**
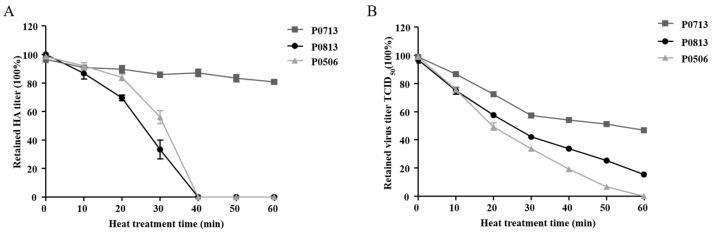
Results from thermostability test of genotype VI NDV. Allantoic fluids of P0506, P0713, and P0813 were treated at 56 °C for 60 min. The HA activity (**A**) and TCID_50_ (**B**) of each virus were determined at 10 min intervals. Means and standard deviations (SD) are shown for three independent experiments.

Since all genotype VI NDVs had the potential molecular features related to thermostability of NDV, the heat-resistant characteristic of genotype VI NDV was investigated. In view of that, VI.2.1.1.2.1 and VI.2.1.1.2.2 were the two most prevalent sub-genotypes in China since about 2007 [13]. Isolates belonging to VI.2.1.1.2.1 (P0813) and VI.2.1.1.2.2 (P0713 and P0506) were selected for the thermostability test. The results showed that the HA titer of P0713 was not significantly changed when treated at 56 °C within 60 min, while both P0813 and P0506 had completely lost their HA activities at 40 min (Figure 3A). Furthermore, the effect of heat treatment on the infectivity of these viruses was examined by performing a TCID_50_ assay. As shown in Figure 3B, P0713 and P0813 retained about 50% and 15% infectivity at 60 min, respectively, while the infectivity of P0506 was completely lost at this time point. According to the criteria for the thermostability of the NDV strains [40], the viruses that can maintain HA activity and infectivity at 56 °C for at least 30 min are considered thermostable strains. Thus, P0813, P0713, and P0506 were thermostable strains, although P0713 was more thermostable than P0813 and P0506. Relatively, the infectivity of P0506 was more sensitive to heat treatment. The amino acid differences in HN protein and the resulting differences in protein structural stability might contribute to the different heat resistance of these viruses.

### 3.5. Identification of Key Amino Acid Residues Involved in Thermostability of HN Protein

**Figure 4:**
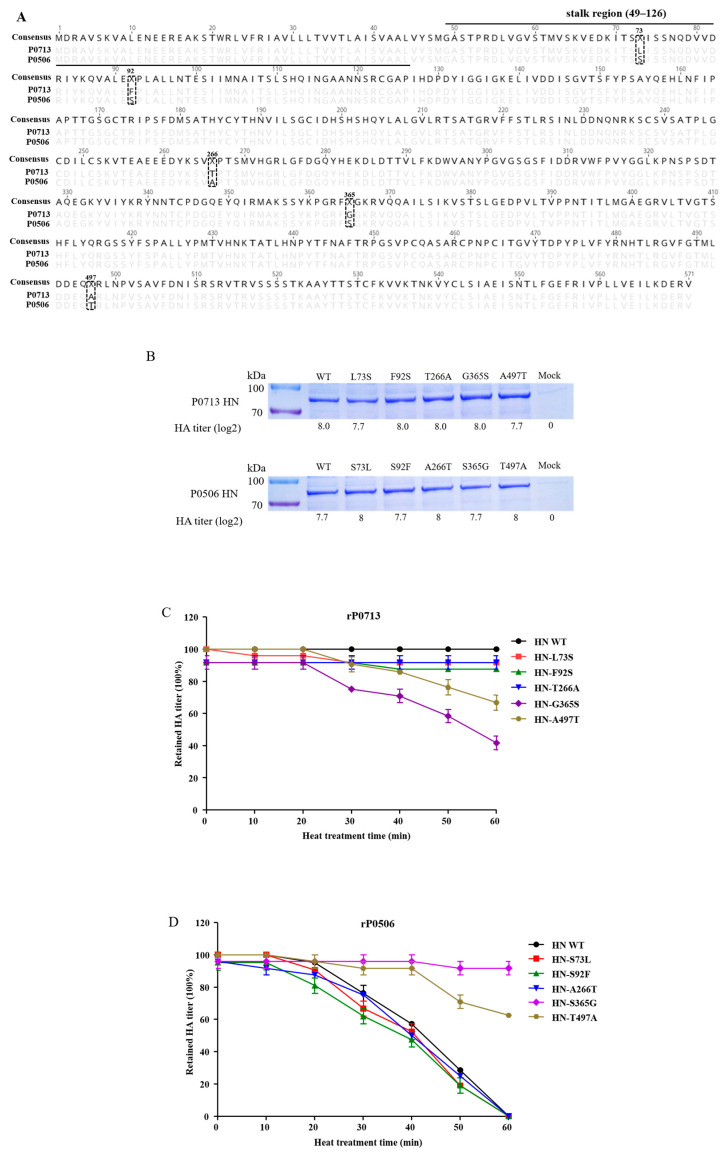
Amino acid residues at 365 and 497 in HN protein involved in its heat resistance. (**A**) Amino acid sequence alignment of HN protein from P0713 and P0506. The different amino acids were labelled by black dashed box. (**B**) Identification of the expressed WT and mutant HN proteins of P0713 and P0506. The purified proteins were examined by SDS-PAGE and HA assay. (**C**,**D**) Results of the thermostability test of WT and mutant HN proteins of P0713 and P0506, respectively. The proteins were treated at 56 °C for 60 min. The HA activity of each protein was determined at 10 min intervals. Means and standard deviations (SD) are shown for three independent experiments.

According to the results of the thermostability test, P0713 and P0506, belonging to sub-genotype VI.2.1.1.2.2, demonstrated distinct resistance to heat treatment. Since HN protein is considered the key determinant of NDV thermostability [27], the amino acid sequences of HN protein from P0713 and P0506 were compared. Multiple amino acid substitutions that were distributed across the whole HN protein, including the stalk region (49–126 aa) and globular head domain (127–571 aa), were found between the two viruses. Specifically, five amino acid substitutions at positions 73, 92, 266, 365, and 497 were found in HN proteins between P0713 and P0506 (Figure 4A).

To investigate the amino acid (s) of HN protein involved in the thermostability, wild-type (WT) HN proteins of P0713 and P0506, as well as mutants with substitutions at 73, 92, 266, 365, and 497, were expressed in Expi293F cells. After purification, the HA assay of HN WT, -L73S, -F92S, -T266A, -G365S, -A497T mutants of P0713 and HN WT, -S73L, -S92F, -A266T, -S365G, -T497A mutants of P0506 was performed and all WT and mutant HNs presented HA activities with HA titer 2^7.7^ to 2^8^. Subsequently, these proteins were examined by SDS-PAGE. All proteins presented bands at about 75 kDa, consistent with the theoretical size of the HN protein. Thus, the WT and mutant HNs were successfully obtained (Figure 4B and Supplementary Figure S1). Then, the HA activity of these proteins after 56 °C heat treatment was examined. As shown in Figure 4C, the HA titers of WT HN of P0713 and HN-L73S, -F92S, -T266A mutants were stable within 60 min, whereas the HA titers of HN-G365S and HN-A497T were decreased after treatment for 30 min and retained about 42% and 67% activities at 60 min, respectively. Moreover, the HA titers of WT HN of rP0506 and HN-S73L, -S92F, -A266T mutants were gradually decreased with time and completely lost HA activities at 60 min, whereas the HN-S365G and HN-T497A mutants retained about 90% and 62% activities at 60 min, respectively (Figure 4D). The findings suggested that amino acid residues at both positions 365 and 497 in the HN protein are involved in its heat resistance.

### 3.6. Residue 365 in HN Protein Significantly Affected the Viral Thermostability

To further validate the effect of residues 365 and 497 in HN protein on the thermostability of P0713 and P0506, recombinant viruses rP0713, rP0713-G365S, -A497T, and rP0506, rP0506-S365G, -T497A were recovered. It demonstrated that there were no significant differences in HA, TCID_50_, and EID_50_ titers between parental P0713 and the rP0713, rP0713-G365S, -A497T viruses, as well as between parental P0506 and the rP0506, rP0506-S365G, -T497A viruses (Table 1), indicating that the mutant at positions 365 and 497 in HN protein did not affect the replication capacities of P0713 and P0506.

**Figure 5:**
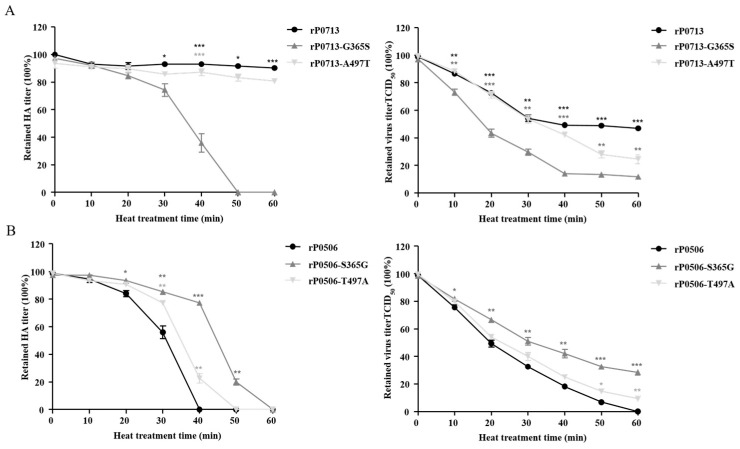
Effects of amino acid residues at positions 365 and 497 in HN protein on thermostability of virus. (**A**) Results of the thermostability test of rP0713, rP0713-G365S, -A497T. (**B**) Results of the thermostability test of rP0506, rP0506-S365G, -T497A. Allantoic fluids of each virus were treated at 56 °C for 60 min. The HA activity and TCID_50_ of each virus were determined at 10 min intervals. Means and standard deviations (SD) are shown for three independent experiments. The retained HA or virus titers among different viruses were analyzed with one-way analysis of variance (*, *p* < 0.05; **, *p* < 0.01; ***, *p* < 0.001).

The G365S and A497T mutations in rP0713-G365S and rP0713-A497T resulted in the loss of HA activity and infectivity at different levels compared to those of the parental rP0713, in which rP0713-G365S showed dramatically decreased thermostability (Figure 5A). In contrast, the S365G and T497A mutations in rP0506-S365G and rP0506-T497A reduced the loss of HA activity and infectivity compared to the parental rP0506. Notably, rP0506-S365G retained higher levels of HA activity and infectivity at the indicated time points (Figure 5B). These results suggested that amino acid residues at positions 365 and 497 in the HN protein were responsible for the thermostability of NDV, in which residue 365 was a crucial determinant.

### 3.7. Substitution Analysis of Residues 365 and 497 in HN Protein

**Figure 6:**
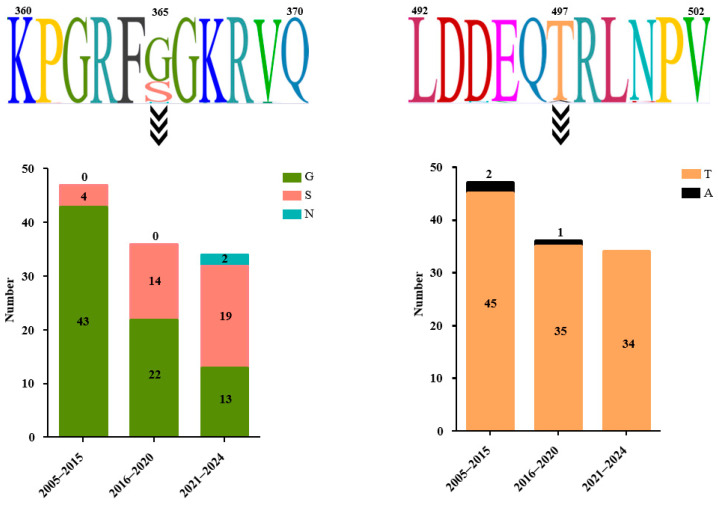
Signature features of amino acid residues at positions 365 and 497 in HN protein from sub-genotype VI.2.1.1.2.2 NDV. Deduced amino acid residues flanking positions 365 and 497 in HN protein were aligned. Amino acid type and number among isolates during different time periods are shown.

To investigate whether the dynamic substitution of residues 365 and 497 in HN protein occurred during the transmission of sub-genotype VI.2.1.1.2.2 NDV, the amino acid sequences of the HN proteins from viruses isolated from different time periods were compared (Figure 6). For residue 365, 43 isolates (91.49%) had G and 4 isolates (8.51%) had S in viruses isolated between 2005 and 2015, 22 isolates (61.11%) had G and 14 isolates (38.89%) had S between 2016 and 2020, and 13 isolates (38.24%) had G, 19 isolates (55.88%) had S, and 2 isolates (5.88%) had N between 2021 and 2024. For residue 497, 45 isolates (95.74%) had T and 2 isolates (4.26%) had A in viruses isolated between 2005 and 2015, 35 isolates (97.22%) had T and 1 isolate (2.78%) had A between 2016 and 2020, and all 34 isolates had T (100%) between 2021 and 2024. These results indicated that residue 497 was conserved in HN protein from sub-genotype VI.2.1.1.2.2 NDV, while residue 365 presented high diversity with time.

### 3.8. Thermostability of Different NDV Strains

**Figure 7:**
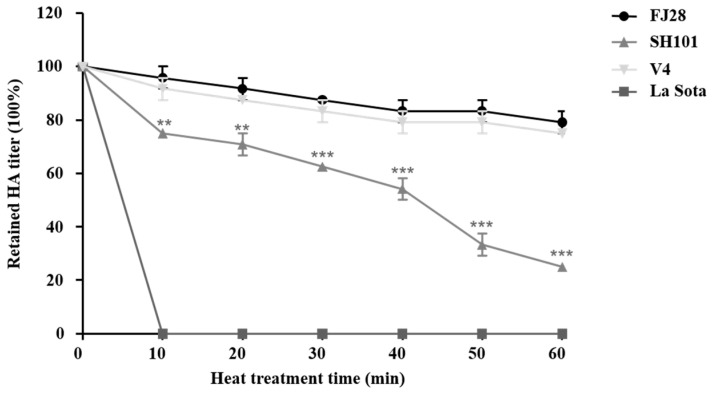
Results from thermostability test of V4, La Sota, FJ28, and SH101. Allantoic fluids of four viruses were treated at 56 °C for 60 min. The HA activity of each virus was determined at 10 min intervals. Means and standard deviations (SD) are shown for three independent experiments. The retained HA titer among different viruses was analyzed with one-way analysis of variance (**, *p* < 0.01; ***, *p* < 0.001).

To investigate whether the G365 in HN protein was a unique molecular marker of thermostability of genotype VI NDV, heat resistance of four strains belonging to different genotypes but with identical G365 in HN protein was determined. As shown in Figure 7, the HA titer of V4 was not significantly changed when treated at 56 °C within 60 min, while La Sota completely lost HA activity at 10 min. In addition, though both FJ28 and SH101 retained HA activities within 60 min, the HA activity retained by FJ28 was significantly higher than that of SH101 at each time point.

## 4. Discussion

Since the first report of genotype VI NDV in the 1970s, this virus has spread rapidly throughout the world and resulted in the third ND pandemic [11], and the epidemiological connections among different continents and hosts have been reported [41]. However, the mechanism of transmission of genotype VI NDV at long distances has not been disclosed. Especially, whether the resistance of NDV to heat contributes to the spread of this virus remains unknown, although it has been suggested that the resistance ability of the virus to environmental factors is beneficial for its inter-host and trans-regional transmission of the virus [8].

In this study, the heat-resistant characteristics of NDV isolates belonging to VI.2.1.1.2.1 (P0813) and VI.2.1.1.2.2 (P0713 and P0506) were evaluated. We found that all three viruses retained HA activity and infectivity after being treated at 56 °C for 30 min, suggesting that all of them can be considered as thermostable strains based on the criteria for the thermostability of the NDV strains [40]. However, P0713 was more thermostable than P0506 and P0813. Since all three viruses contained residues P315 and V369 in the HN protein, which have been suggested as the key residues for thermostability of genotype VIII thermostable HR09 strain, we inferred that there should be other amino acid residues responsible for the thermostable phenotype of these viruses. Although HN is identified as the crucial thermostable determinant, the association of F protein with the thermostability of NDV has also been reported [26,27]. Four different amino acids were found between F proteins of P0713 and P0506, three of them (position 2, 13, 25) were located at signal peptide region, and one (position 530) were located in cytoplasmic tail region, suggesting that F protein should not be involved in the difference in thermostability of P0713 and P0506. In contrast, residues at positions 365 and 497 in the HN protein were involved in the thermostability of P0703, and G365 was identified as a key thermostable determinant. Furthermore, the residues at both positions 365 and 497 in the HN protein of V4, La Sota, FJ28, and SH10 were G and A, but distinct thermostable phenotypes of these strains were demonstrated, indicating a unique mechanism of thermostability of genotype VI NDV. Due to residue 365 located at the globular head domain of HN protein that is responsible for receptor binding, whether this residue contributes to the viral thermostable phenotype by affecting the stability of the spatial structure of the receptor binding region remains to be further studied. Moreover, thermostable V4 and FJ28 were avirulent strains, while HR09 was a virulent strain. In addition, P0713, P0506, and P0813, which possessed identical virulent F protein cleavage site motif ^112^RRQKRF^117^, demonstrated different resistance to heat treatment. Collectively, these findings suggested that the residue at position 365 in the HN protein and the thermostability might not be involved in the pathogenicity of NDV, as previously reported [40].

So far, the known thermostable NDV strains were mainly isolated from the tropical zone (I V4, I-2, and AF2240) or wild birds (K148/08) [20,21,22,23]. Recently, we have reported the widespread transmission and prevalence of class I NDV in live poultry markets in China [33]. Notably, most of the class I NDV isolated from birds or environmental samples in live poultry markets have also presented a thermostable phenotype (our unpublished data). The thermostability of class I NDV may be one of the reasons for the widespread circulation of the virus. This speculation might also be appropriate for explaining the transcontinental transmission of genotype VI NDV.

Genetic evolution analysis suggested that Europe was the epicenter of genotype VI NDV and the sub-genotype VI.2.1.1.2.2 NDV in China should originate from Belgium isolates [13]. Amino acid sequence analysis showed that both residues G365 and A497, that was responsible for the thermostability of P0713, were present in the HN protein from a Belgium sub-genotype VI.2.1.1.2.2 strain (Belgium PPMV-1/Belgium/11-07574/2011). We inferred that this Belgium strain may be the ancestor of P0713-like sub-genotype VI.2.1.1.2.2 NDV in China. Especially, the thermostable phenotype determined by G365 and A497 should contribute to the long-distance transmission of this virus from Belgium to China. In addition, A497 was found in 3 of 117 sub-genotype VI.2.1.1.2.2 isolates, while G365 was found in 78 of 117 sub-genotype VI.2.1.1.2.2 isolates. This was consistent with the result that although both G365 and A497 were involved in the thermostability of P0713, residue 365 was a crucial determinant. Another interesting finding was that the proportion of viruses containing G365 gradually decreased over time. The widespread transmission and circulation of sub-genotype VI.2.1.1.2.2 NDV worldwide may lead to this virus more easily to contact with susceptible animals; thus, there is no need to maintain the thermostable phenotype as suggested previously [8]. This needs to be further elucidated.

The genotype VI strains in class II are economically important causative agents for the poultry industry and are involved in most current outbreaks of ND across the world. Since its first emergence, sub-genotype VI.2.1.1.2.2 NDV has been the most prevalent sub-genotype worldwide. Due to a lack of available vaccines, viruses of this genotype are still circulating in pigeon flocks and pose a potential threat to domestic poultry. The findings in the present study provided insight for understanding the mechanism of transmission of genotype VI NDV. The identification of the key amino acid residue that is responsible for the thermostability is beneficial for the rational development of live thermostable genotype VI NDVs [42,43,44,45].

## Figures and Tables

**Table 1:** Titers of rescued viruses.

Virus	HA (log_2_)	TCID_50_ (log_10_/0.1 mL)	EID_50_ (log_10_/0.1 mL)
P0713	8	10^7.6^	10^8.3^
rP0713	8	10^7.7^	10^8.5^
rP0713-G365S	8	10^7.5^	10^8.4^
rP0713-A497T	8	10^7.7^	10^8.5^
P0506	8	10^8.0^	10^8.5^
rP0506	8	10^7.9^	10^8.3^
rP0506-S365G	8	10^8.1^	10^8.4^
rP0506-T497A	8	10^7.8^	10^8.4^

## Data Availability

The original contributions presented in the study are included in the article; further inquiries can be directed to the corresponding authors.

## Supplementary Materials

The following supporting information can be downloaded at: https://www.mdpi.com/article/10.3390/v17070977/s1, Figure S1: The original gel image of Figure 4B.
