# Supplementary figures and images for: Residue 365 in Hemagglutinin–Neuraminidase Is a Key Thermostable Determinant of Genotype VI.2.1.1.2.2 Newcastle Disease Virus
Source: Viruses. 2025 Jul 13;17(7):977. doi: 10.3390/v17070977 (PMC12300083; doi:10.3390/v17070977)

### P0713 HN

|                     | WT    | L73S  | F92S  | T266A | G365S | A497T | Mock |
|---------------------|-------|-------|-------|-------|-------|-------|------|
| percentage of HN(%) | 90.65 | 90.46 | 90.05 | 90.11 | 90.26 | 90.40 |      |

kDa

170  
130  
100  
70  
55  
40  
35  
25  
15

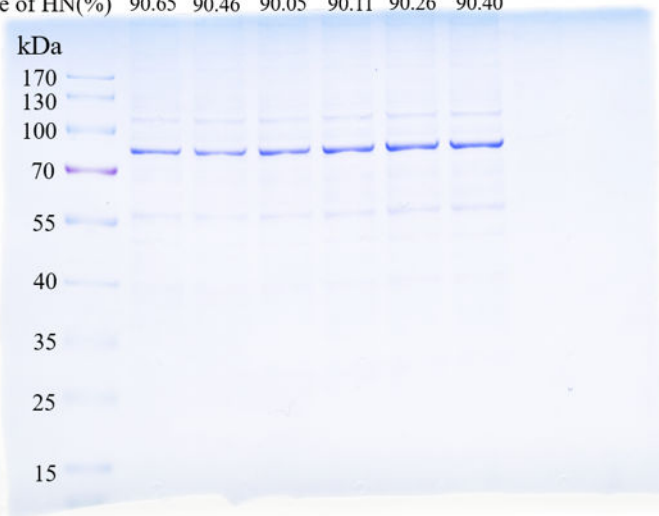

### P0506 HN

|                     | WT    | S73L  | S92F  | A266T | S365G | T497A | Mock |
|---------------------|-------|-------|-------|-------|-------|-------|------|
| percentage of HN(%) | 90.35 | 90.73 | 90.86 | 90.28 | 90.11 | 90.08 |      |

kDa

170  
130  
100  
70  
55  
40  
35  
15

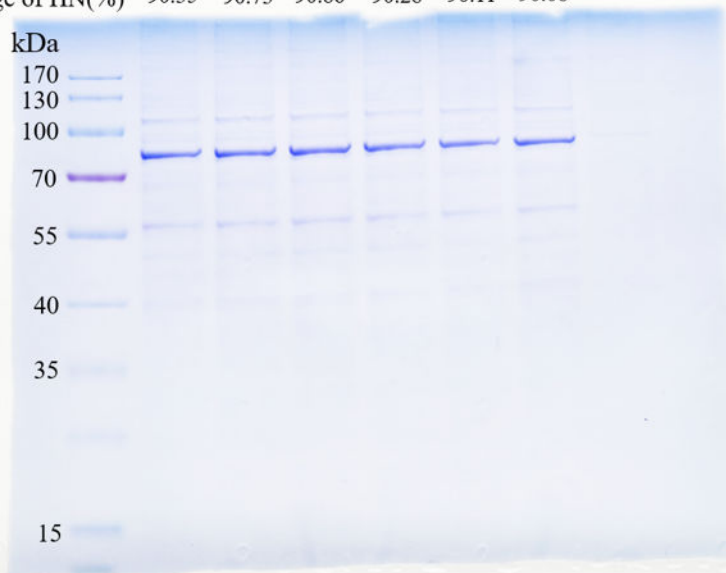

Supplement: Supplementary file 1 [file viruses-17-00977-s001.zip › viruses-3717820-supplementary.pdf]
